# FlexO2: A patient-controlled oxygen flow selector improving autonomy and daily function in long-term oxygen therapy (LTOT)

**DOI:** 10.1186/s12931-025-03274-x

**Published:** 2025-05-26

**Authors:** Michael Runold, Ingegerd Karlsson, Magda Borén

**Affiliations:** 1 Department of Respiratory Medicine and Allergology, Karolinska University Hospital, Solna, 171 64 Sweden; 2 Department of Medicine, Karolinska Institute, Stockholm, Sweden

**Keywords:** Long-term oxygen therapy, Patient autonomy, COPD, Oxygen Titration, Activities of daily living

## Abstract

**Background:**

Chronic respiratory insufficiency associated with severe resting hypoxemia necessitates long-term oxygen therapy (LTOT), yet existing devices often impede daily activities due to cumbersome flow adjustments, increasing reliance on caregivers. FlexO2 is a novel mechanical regulator that enables switching between preset oxygen flow rates for rest and activity. This proof-of-concept study evaluated its impact on patient autonomy, physical activity, and quality of life.

**Methods:**

In a consecutive, non-randomized pre-post intervention proof-of-concept study at Karolinska University Hospital, 26 patients on LTOT (median age 77; 69% COPD) used FlexO2 for three months. The device, worn around the neck, allowed patients to self-adjust oxygen doses without accessing the concentrator. Outcomes included ease of use measured by visual analogue scale (VAS), physical activity levels, COPD Assessment Test (CAT), EQ-5D-5 L index, and frequency of dose adjustments.

**Results:**

Ease of dose adjustment increased from a VAS score of 14 to 92 (*p* < 0.001), with 92% of patients reporting improved ease of adjustment (baseline 7.7%; *p* < 0.001). Daily adjustment frequency doubled (8 to 15; *p* = 0.001). Patient-reported activity capacity improved from a VAS of 11 to 80 (*p* < 0.001). Quality-of-life scores measured by VAS increased from 19 to 61 (*p* < 0.001), while CAT scores decreased from a median of 26.0 to 22.5 (*p* = 0.05). The EQ-5D-5 L index remained stable (0.68 to 0.70; *p* = 0.7), although 38% of patients showed individual improvements. Device usability was high (83% satisfaction), though 15% reported tubing tangling or airflow issues.

**Conclusion:**

FlexO2 significantly improved the ease of oxygen dose adjustment and physical activity capacity, potentially enhancing patient autonomy in LTOT. While overall patient-reported quality-of-life scores improved, objective quality-of-life outcomes remained stable. Further studies are warranted to explore long-term clinical outcomes and the potential impact on caregiver burden.

**Supplementary Information:**

The online version contains supplementary material available at 10.1186/s12931-025-03274-x.

## Take-home message

FlexO2, a patient-controlled oxygen flow selector, enables easy switching between rest/activity doses, improving autonomy, daily function, and quality of life in LTOT patients (*n* = 26). High usability with minimal issues reduces caregiver dependency.

## Introduction

Chronic respiratory diseases are associated with significant morbidity, mortality, and healthcare utilization, leading to a substantial burden on healthcare systems [1].

In the United States, total healthcare spending on respiratory conditions reached approximately $170.8 billion in 2016, reflecting an increase of $71.7 billion since 1996 [2].

Chronic respiratory insufficiency, particularly in conditions such as severe Chronic Obstructive Pulmonary Disease (COPD) and interstitial lung diseases (ILD), often leads to severe hypoxia, necessitating home long-term oxygen therapy (LTOT) [3, 4]. Although early landmark studies indicated that continuous LTOT could triple survival for COPD patients with severe chronic hypoxia [5, 6], it is important to acknowledge that these investigations were limited by relatively small sample sizes, which may have overlooked broader quality-of-life outcomes.

Epidemiological data from Sweden illustrate the prevalence and significance of LTOT. Approximately 2,596 patients received LTOT, representing a national prevalence of 31.6 per 100,000 inhabitants. Additionally, adherence to LTOT varies between 45% and 70%, indicating that while many patients benefit from this therapy, compliance remains a challenge [7].

Patients often find themselves tethered to their oxygen devices, which restricts their ability to perform activities of daily living (ADLs) [8, 9].

During physical activity, patients typically experience an increase in oxygen demand. However, many patients may not receive adequate oxygen supplementation during these times if their devices cannot adjust quickly or efficiently to meet these increased needs [10].

Current oxygen devices often lack user-friendly mechanisms for adjusting flow rates on the go. Patients may need to stop their activity to modify their oxygen settings, which can be impractical and frustrating [8].

Caregivers, such as family members or nurses, often play a pivotal role in managing the therapy, including monitoring oxygen levels, adjusting flow rates, and assisting with mobility to the concentrator [11]. Patients may also lack the physical strength to reach the equipment themselves, requiring caregivers to respond promptly to their needs [11–14].

The physical and emotional burden on caregivers increases as they must continuously ensure the equipment’s proper functioning while balancing their daily responsibilities. This often leads to stress and caregiver fatigue, particularly in cases where LTOT requires frequent adjustments [11–14].

This study aimed to evaluate the impact of FlexO2 on autonomy, daily physical function, and quality of life in patients on LTOT. We investigated whether simplifying flow adjustments could improve self-management of oxygen therapy during routine activities.

## Methodology

### Design

This research is a consecutive, non-randomized, pre-post-intervention proof-of-concept study designed to evaluate the efficacy and safety of the FlexO2 device in patients receiving long-term oxygen therapy (LTOT). The trial was conducted at the Karolinska University Hospital, Faculty of Medicine, Department of Respiratory Medicine and Allergology, Solna, Stockholm.

### Participants

Thirty patients were recruited from the regular flow of patients at the Department of Respiratory Medicine, of whom twenty-six met the inclusion criteria.

The inclusion criteria were patients with chronic respiratory insufficiency receiving long-term oxygen therapy (LTOT) for at least six months at two different flow settings (at rest and during activity) and the ability to understand the study procedures and provide informed consent.

Exclusion criteria included patients with increased PCO2 above 6 kPa or 45 mmHg, language difficulties or reduced cognitive ability and those who had recently initiated oxygen therapy.

### Intervention and control

**Fig. 1:**
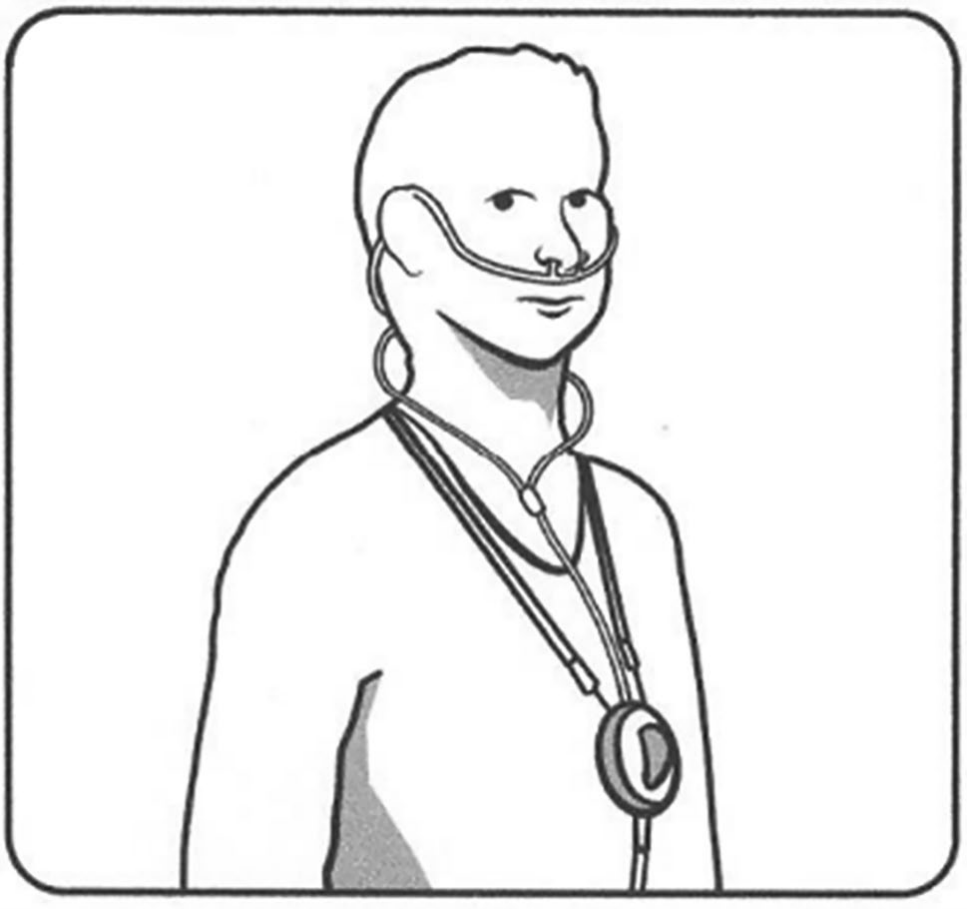
Illustration showing the FlexO2 oxygen flow control device worn around the neck, with nasal cannula in place

FlexO2 is a patient-controlled oxygen flow selector that allows the user to quickly switch between two preset oxygen doses for activity and rest. The device was connected to the patient’s nasal cannula, allowing them to modify their oxygen dose without needing to access the oxygen concentrator. The two doses are preset by healthcare professionals based on prescribed oxygen flow. FlexO2 is worn around the neck (Fig. 1), relieving the patient’s ears from strain. It is connected at the bottom of the valve to the tube from the oxygen concentrator and on top of the nasal cannula [15]. The device is currently CE marked as a Class IIa medical device. Is is approved under the Medical Devices Directive (MDD 93/42/EEC), and has a valid exemption while the MDR certification process is ongoing.

All participants received the FlexO2 device. The patient’s oxygen dose at rest was determined based on an arterial blood gas analysis, while the dose during activity was established using the six-minute walk test (6MWT). The oxygen dose for each setting was initially determined and prescribed by a physician and then calibrated by an oxygen nurse according to the patient’s needs as part of their usual care. Each month, the device was replaced with a new one.

A within-subject design was implemented, where each participant served as their control. Outcomes were compared at enrollment and after three months of FlexO2 usage to gauge the intervention’s impact. This approach allowed for direct assessment of the device’s effect on each patient. Patients were instructed to return to traditional oxygen flow adjustment as a contingency measure in case of problems or device malfunction. An oxygen therapy nurse conducted telephone follow-ups to check on the device’s function.

### Data collection and instruments

Interviews and structured questionnaire forms were used to assess the function of the oxygen regulator and related questions. Data was collected from participants at baseline and after three months, including (1) demographic data, (2) clinical data related to the use and handling of the FlexO2 device, and (3) quality of life and health assessments using the EQ-5D questionnaire and the COPD Assessment Test (CAT) [17, 18].

Participants were scheduled for nurse visits during which practical handling tests were conducted. During these visits, an oxygen therapy nurse observed each participant’s ability to adjust the oxygen dose directly on the concentrator and remotely using the FlexO2 device. The nurse noted any difficulties or errors (e.g., challenges in adjusting the dose, tubing tangling, or airflow issues), and provided on-site guidance when necessary.

To complement the in-person assessments, telephone follow-ups were conducted periodically by the oxygen therapy nurse. These calls served to ensure the device was functioning correctly, to answer participant queries, and to record any issues or adverse events reported between the scheduled visits.

### Outcomes

The primary outcomes were:

(A) Overall quality of life - assessed using Visual Analogue Scale (VAS, 0-100).
(B) Ability to be active at home - assessed using Visual Analogue Scale (VAS, 0-100).
(C) Ease of adjusting oxygen dose - assessed using Visual Analogue Scale (VAS, 0-100).
(D) Daily frequency of oxygen dose adjustments - assessed by patient-reported counts.

The secondary outcomes focused on oxygen dose adjustments, specifically the frequency and ease of adjustments in several different situations, along with overall patient satisfaction and the user-friendliness of the FlexO2 device. Secondary outcomes also include EQ-5D and the COPD Assessment Test (CAT).

Patients were asked about their overall satisfaction with FlexO2 and to report any problems or device-related safety issues experienced during the three-month study period.

All outcomes were measured at baseline and at the 3-month follow-up to evaluate changes associated with FlexO2 use.

### Statistical analyses

Descriptive statistics were used to summarize baseline characteristics. Continuous variables were expressed as medians with interquartile ranges (IQRs) due to the non-parametric nature of the data.

A minimum important difference of 2 points was used to assess changes in the CAT score [19].

To convert the EQ-5D-5 L to an index, the guidelines established in the relevant literature were followed, utilizing country-specific value sets. For this purpose, the most recent study conducted in Sweden by Sun et al. (2022) was referenced [20].

The minimally important difference (MID) was applied to evaluate whether changes in scores were clinically significant. In the context of chronic obstructive pulmonary disease (COPD) and other respiratory diseases, a MID of 0.051 for utility indices and 6.9 points for Visual Analogue Scale (VAS) scores are considered clinically important differences, as suggested by Nolan et al. [21].

The Wilcoxon signed-rank test was used to compare paired continuous variables, and McNemar’s test was used for paired categorical data. A p-value of less than 0.05 was considered statistically significant. Data was visualized using dot plots, with the median as the center dot and Q1 and Q3 as the lower and upper whiskers. All statistical analyses were done using R (version 4.4.0) [22].

### Data availability declaration

The data supporting the findings of this study will be made available upon request.

### Clinical trial number

Clinical trial number is not applicable. The study was registered locally in Stockholm. As it was not initially listed on clinicaltrials.gov, retrospective registration was not possible.

### Ethics, consent to participate, and consent to publish

 Ethical approvalwas obtained from the Regional Ethical Review Board in Stockholm (EPN 2017/673 − 31/1). The trial was conducted during the period of September 2017 to November 2022.

Written informed consent was obtained from all participants before their inclusion in the study, covering their participation and the use of their data for publication. All procedures were performed in accordance with the ethical standards of the institutional and national committees and with the Helsinki Declaration [16].

### Funding declaration

This study was supported by the Swedish Heart-Lung Foundation (Hjärt-Lungfonden). FlexO2 devices were provided by Lungflex AB, Ljungby, Sweden at no cost. Neither the funding body nor the device manufacturer participated in the study design, data collection, analysis, interpretation, or manuscript preparation.

## Results

### Patient baseline characteristics

The study included 26 participants (18 females and 8 males). The age range was 55–88, with a median age at study entry of 77 years (IQR: 74, 83). The median duration of LTOT use before the study was 20 months (IQR: 16, 36). The patients’ primary indication for LTOT treatment were COPD (17 patients), idiopathic pulmonary fibrosis (6 patients), and pulmonary arterial hypertension (3 patients).

At baseline, participants reported using oxygen for a median of 24.0 h per day (IQR: 22.0, 24.0), which remained unchanged at the 3-month follow-up. The median prescribed oxygen dose at rest was 2.0 L/min (IQR: 1.5, 3.0) at both time points. The prescribed oxygen dose during activity increased from a median of 4.0 L/min (IQR: 3.0, 6.0) at baseline to a median of 5.0 L/min (IQR: 4.00, 6.00) after 3 months (*p* = 0.008).

### Primary outcomes

**Fig. 2:**
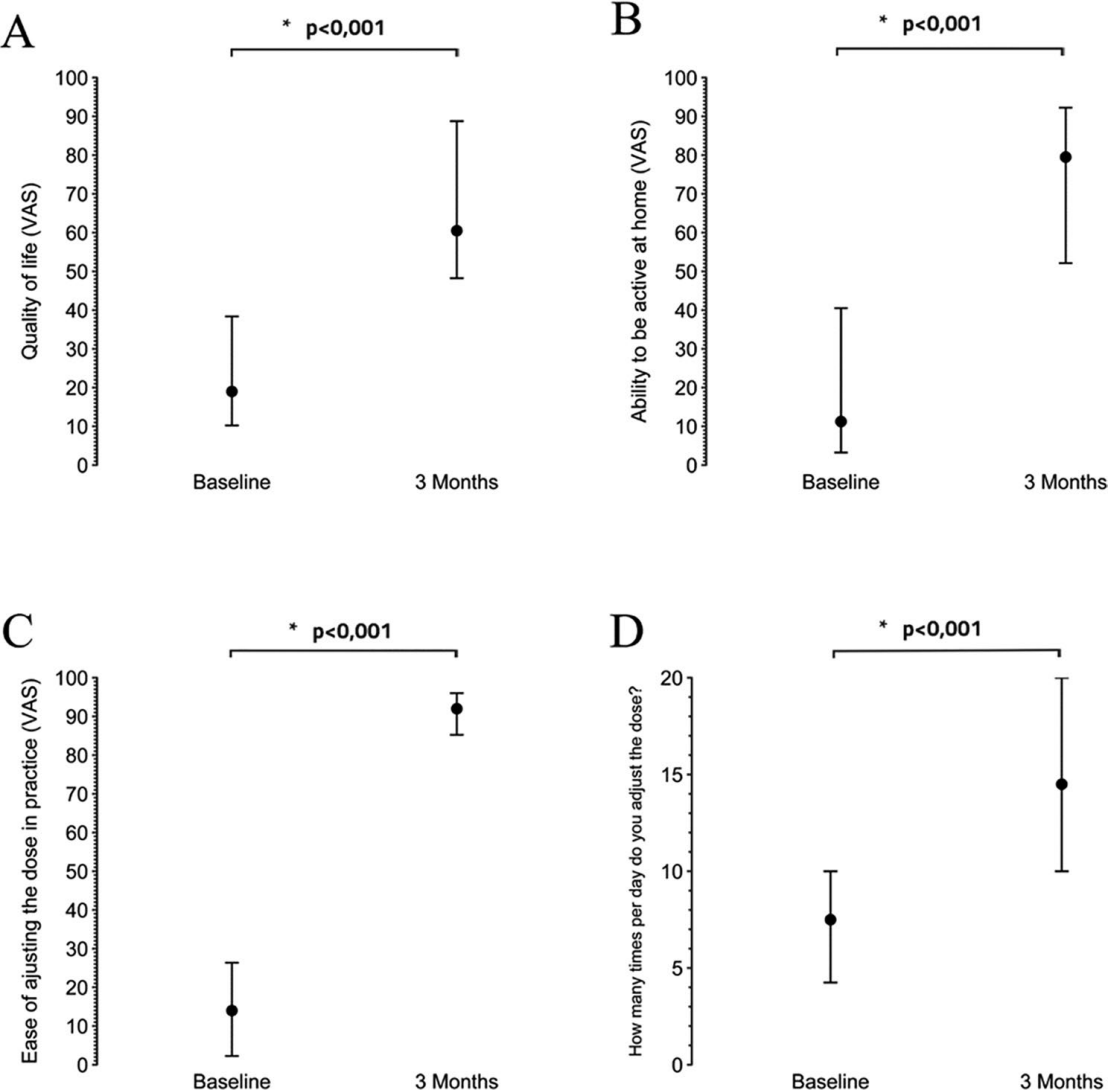
The figure shows the changes in primary outcome measures from baseline to 3-month follow-up after using the FlexO_2_ device. The point represents the median, the lower and upper whiskers represent Q1 and Q3 respectively. **A**; Quality of life, **B**; ability to be active at home, **C**; ease of adjusting oxygen, **D**; the daily frequency of oxygen dose adjustments

The study showed significant changes across all primary outcome measures after 3 months of FlexO2 regulator use. The ease of adjusting oxygen dose in practice increased significantly from a baseline VAS score of 14 (2, 27) to 92 (85, 96) (*p* < 0.001). The proportion of patients finding it easy to adjust dose during activity improved from 7.7% (*n* = 2) at baseline to 92% (*n* = 24) (*p* < 0.001) after 3 months. Daily frequency of oxygen dose adjustments before activities increased from 8 (4, 10) to 15 (10, 20) times (*p* = 0.001). The self-reported ability to be active at home improved from a VAS score of 11 (3, 44) to 80 (52, 93) (*p* < 0.001), while the quality-of-life scores increased from 19 (10, 40) to 61 (48, 89) (*p* < 0.001). The results are summarized in (Fig. 2).

### Secondary outcomes

The median number of daily oxygen dose adjustments during activities increased after 3 months with FlexO2. Significant increases in switching oxygen doses between rest and activity were seen when getting out of bed (31–81%, *p* < 0.001), getting up from sitting to standing (15–62%, *p* = 0.002), moving around at home (40–88%, *p* = 0.003), dressing and undressing (31–88%, *p* < 0.001), doing household chores (50–95%, *p* = 0.013), having meals (8.3–46%, *p* = 0.027), with other physical activity (57–86%, *p* = 0.023), and with shortness of breath (54–92%, *p* = 0.008). (Table S1)

The proportion of participants who found it easy to follow the prescribed oxygen dose increased from 54% at baseline to 85% after 3 months with FlexO2 (*p* = 0.043). (Table S2)

The median CAT-5 score was 26.0 (19.0, 28.0) at baseline and 22.5 (19.0, 29.0) at 3 months (*p* = 0.05). Individual analysis of CAT-5 scores revealed that 58% of patients (*n* = 15) demonstrated clinically important improvement, while 15% (*n* = 4) showed deterioration. The remaining 27% (*n* = 7) maintained stable scores with no clinically important change.

The median EQ-5D-5 L converted index score was 0.68 (0.50, 0.82) at baseline compared to 0.70 (0.55, 0.83) at 3 months (*p* = 0.7). Individual analysis of EQ-5D-5 L converted index scores revealed that 10 patients (38%) showed clinically important improvement, and 10 patients (38%) showed deterioration, while 6 patients (23%) maintained stable scores with no clinically important change.

Most of the participants 83% (*n* = 20) found the FlexO2 device easy to carry and use, meanwhile, 15% (*n* = 4) reported experiencing problems with the device but no harm to the patients was encountered. One participant noted insufficient airflow on the lower setting and kept the device on the higher setting; the other three issues were tangled tubing. In two of these cases, the tangling involved the longer tubing connecting the oxygen concentrator to the FlexO2, while the last mentioned “tangling tubes.”.

## Discussion

The FlexO2 device addresses a critical limitation in traditional oxygen therapy by enabling personalized oxygen delivery that adapts to patients’ dynamic physiological needs. Oxygen requirements naturally fluctuate throughout the day, varying with different activity levels and during sleep [23]. This should be addressed during LTOT as recommended by The British Thoracic Society (BTS) [24].

The primary outcomes revealed a substantial increase in the ease of adjusting oxygen doses during daily activities, with the proportion of patients finding it easy to adjust doses rising from 7.7 to 92%. This improvement is clinically significant, as inadequate oxygen titration during physical activity is a common challenge for patients on LTOT and can lead to reduced physical activity and worsening quality of life [25, 26]. The increased ease of oxygen dose adjustment can also have broader implications, such as improving autonomy and reducing the reliance on next of kin for assistance; it has the potential to minimize the risk of complications due to inadequate oxygenation during activity, potentially preventing injuries and reducing the need for hospital care.

Physical activity is crucial for patients with chronic hypoxemia, directly impacting their quality of life and overall health, which is limited in patients on LTOT [26–28]. The FlexO2 device enabled patients to confidently initiate and sustain physical activity by improving their ability to engage in various home activities and making oxygen flow rate adjustments easier.

Newer solutions, like the current physiological closed-loop oxygen therapy systems, rely on continuous pulse oximeter monitoring, which is problematic due to motion artifacts and delayed respiratory change detection. These complex and costly systems have limited practical use for home therapy [29, 30]. In contrast, FlexO2 offers a straightforward solution to manually adjust the flow rate according to the activity without continuous monitoring. The increase in the proportion of patients who found it easy to follow prescribed oxygen doses (from 54 to 85%) further supports the usability and effectiveness of the FlexO2 device in clinical practice [25, 27, 31, 32]. Moreover, adjustments of oxygen flow on stationary concentrators generally mean that the patient must adjust the flow rate on a rotameter. This is often cumbersome for the patient and may result in a flow setting above or below the prescribed flow [33, 34].

As assessed by the CAT-5 and EQ-5D-5 L scores, quality-of-life measures showed mixed results. While the median CAT-5 score improved slightly, individual analysis revealed that 58% of patients experienced clinically important improvements, suggesting that the FlexO2 regulator may benefit a subset of patients more significantly. The EQ-5D-5 L index scores did not show a significant overall change; however, they demonstrated equal proportions of patients’ improvement and deterioration. This variability may reflect the diverse clinical profiles and comorbidities of the study population and the subjective nature of quality-of-life assessments [33].

The high acceptability of the FlexO2 device, with 83% of participants finding it easy to carry and use, is a notable strength of this study. However, the reported issues with tangled tubing and insufficient airflow in a small subset of users highlight areas for potential improvement in device design. These findings are consistent with previous studies that have identified practical challenges, such as tubing management and device portability, as barriers to effective oxygen therapy [26, 35].

### Limitations

Our study has several limitations that should be considered when interpreting the results. The absence of a control group limits our ability to distinguish the specific effects of the FlexO2 device from confounding factors such as disease progression and placebo effects. The reliance on subjective patient-reported outcomes, without objective measures like step counts, is another limitation. The generic quality-of-life questionnaire used may lack sensitivity in detecting meaningful changes in this patient population.

With a small sample size (*n* = 26), subgroup analyses by age, COPD status, or baseline activity were not feasible due to statistical power concerns. Our conclusions are primarily limited to the ease and frequency of oxygen dose adjustments. While improvements in autonomy and physical activity were observed, safety was not formally assessed, and quality-of-life improvements should be interpreted cautiously.

Data on caregiver burden and experience were not collected, which is an important aspect of long-term oxygen therapy management.

The FlexO2 device has technical constraints that warrant consideration in clinical implementation. It has been tested to withstand oxygen source pressures up to 1.5 bar; beyond this threshold, the connections may become unstable with risk of disconnection. Additionally, as noted by some participants, issues with tubing management occurred in 15% of cases, suggesting potential design improvements.

Future studies should address these limitations by incorporating control groups, objective physical activity measures, and disease-specific quality-of-life assessments. Additionally, exploring caregiver perspectives and long-term device performance will provide a more comprehensive understanding of the FlexO2 device’s impact and feasibility in clinical practice.

## Conclusions

The FlexO2 device addresses a critical challenge in long-term oxygen therapy (LTOT) by enabling patients to easily adjust their oxygen flow between rest and activity settings. Our findings demonstrate that patients using FlexO2 experienced significantly improved ease of oxygen adjustment. The frequency of daily flow adjustments doubled, indicating patients could better match oxygen delivery to their moment-to-moment needs throughout daily activities like getting out of bed, standing up, and performing household chores.

These improvements in oxygen management were accompanied by high usability ratings, with most participants finding the device practical for daily use.

This study provides promising initial evidence that the FlexO2 device offers a practical solution for dynamic oxygen adjustment in LTOT. Further research with larger sample sizes, control groups, and longer follow-up periods remains necessary to definitively establish its clinical utility, durability, and impact on objective health outcomes.

## Electronic supplementary material

Below is the link to the electronic supplementary material.

Supplementary Material 1

## Data Availability

The data supporting the findings of this study will be made available upon request.

## References

[1] Momtazmanesh S, Moghaddam SS, Ghamari SH et al. Global burden of chronic respiratory diseases and risk factors, 1990–2019: an update from the Global Burden of Disease Study 2019. EClinicalMedicine [Internet] Elsevier Ltd. 2023 [cited 2024 Nov 23];59. Available from: http://www.thelancet.com/article/S258953702300113X/fulltext 10.1016/j.eclinm.2023.101936 PMC7614570 37229504

[2] Duan KI, Birger M, Au DH et al. Health care spending on respiratory diseases in the United States, 1996–2016. Am J Respir Crit Care Med [Internet] American Thoracic Society. 2023 [cited 2024 Nov 23];207:183–192. Available from: https://pmc.ncbi.nlm.nih.gov/articles/PMC9893322/10.1164/rccm.202202-0294OC PMC9893322 35997678

[3] Adeloye D, Song P, Zhu Y et al. Global, regional, and national prevalence of, and risk factors for, chronic obstructive pulmonary disease (COPD) in 2019: a systematic review and modelling analysis. Lancet Respir Med [Internet] Elsevier Ltd. 2022 [cited 2024 Nov 23];10:447–458. Available from: http://www.thelancet.com/article/S2213260021005117/fulltext 10.1016/S2213-2600(21)00511-7 PMC9050565 35279265

[4] Aida A, Miyamoto K, Nishimura M et al. Prognostic value of hypercapnia in patients with chronic respiratory failure during long-term oxygen therapy. Am J Respir Crit Care Med [Internet] Am J Respir Crit Care Med. 1998 [cited 2024 Nov 23];158:188–193. Available from: https://pubmed.ncbi.nlm.nih.gov/9655728/10.1164/ajrccm.158.1.9703092 9655728

[5] Long term domiciliary oxygen therapy in, chronic hypoxic cor pulmonale complicating chronic bronchitis and emphysema. Report of the Medical Research Council Working Party. The Lancet [Internet]. 1981;317:681–686. Available from: https://www.scopus.com/inward/record.uri?eid=2-s2.0-84921006141&doi=10.1016%2fS0140-6736%2881%2991970-X&partnerID=40&md5=4df8fb709d158c98ca11b90125345507 6110912

[6] Continuous or nocturnal oxygen therapy in hypoxemic chronic obstructive lung disease. Ann Intern Med [Internet]. American college of physicians. 1980;93:391–398. Available from: https://www.acpjournals.org/doi/abs/10.7326/0003-4819-93-3-391 10.7326/0003-4819-93-3-391 6776858

[7] Ekström M, Ahmadi Z, Larsson H et al. A nationwide structure for valid long-term oxygen therapy: 29-year prospective data in Sweden. Int J Chron Obstruct Pulmon Dis [Internet] Dove Press. 2017 [cited 2024 Nov 23];12:3159–3169. Available from: https://www.dovepress.com/a-nationwide-structure-for-valid-long-term-oxygen-therapy-29-year-pros-peer-reviewed-fulltext-article-COPD 10.2147/COPD.S140264 PMC5669791 29133978

[8] Hardavella G, Karampinis I, Frille A et al. Oxygen devices and delivery systems. Breathe [Internet] European Respiratory Society. 2019 [cited 2024 Nov 23];15:e108. Available from: https://pmc.ncbi.nlm.nih.gov/articles/PMC6876135/10.1183/20734735.0204-2019 PMC6876135 31777573

[9] Cani KC, Matte DL, Silva IJCS et al. Impact of home oxygen therapy on the level of physical activities in daily life in subjects with COPD. Respir Care [Internet] Respiratory Care. 2019 [cited 2024 Nov 23];64:1392–1400. Available from: https://rc.rcjournal.com/content/64/11/1392 10.4187/respcare.06206 31138730

[10] Koczulla AR, Schneeberger T, Jarosch I et al. Long-term oxygen therapy: current evidence and practical, day-to-day considerations. Dtsch Arztebl Int [Internet] Deutscher Arzte-Verlag GmbH. 2018 [cited 2024 Nov 23];115:871. Available from: https://pmc.ncbi.nlm.nih.gov/articles/PMC6381774/10.3238/arztebl.2018.0871 PMC6381774 30765024

[11] Sami R, Savari MA, Mansourian M et al. Effect of long-term oxygen therapy on reducing rehospitalization of patients with chronic obstructive pulmonary disease: a systematic review and meta-analysis. Pulm Ther [Internet] Pulm Ther. 2023 [cited 2025 Jan 14];9:255–270. Available from: https://pubmed.ncbi.nlm.nih.gov/37093408/10.1007/s41030-023-00221-3 PMC10203089 37093408

[12] Garnet BJ, Jean E, Lankenau RD et al. Identification of male COPD patients with exertional hypoxemia who may benefit from long-term oxygen therapy. PLoS One [Internet] PLoS One. 2023 [cited 2025 Jan 14];18. Available from: https://pubmed.ncbi.nlm.nih.gov/37023024/10.1371/journal.pone.0283949 PMC10079074 37023024

[13] Robinson T. Living with severe hypoxic COPD: the patients’ experience. Nurs Times [Internet]. 2005 [cited 2025 Jan 14];101:38–42. Available from: https://europepmc.org/article/MED/15759524 15759524

[14] Kanervisto M, Kaistila T, Paavilainen E. Severe chronic obstructive pulmonary disease in a family’s everyday life in Finland: perceptions of people with chronic obstructive pulmonary disease and their spouses. Nurs Health Sci [Internet] Nurs Health Sci. 2007 [cited 2025 Jan 14];9:40–47. Available from: https://pubmed.ncbi.nlm.nih.gov/17300544/10.1111/j.1442-2018.2007.00303.x 17300544

[15] Homepage - Flex O2 [Internet]. [cited 2025 Jan 14]. Available from: https://flexo2.com/

[16] Association WM, World medical association declaration of helsinki: ethical principles for medical research involving human subjects. JAMA [Internet]. 2013;310:2191–2194. Available from: 10.1001/jama.2013.281053 10.1001/jama.2013.281053 24141714

[17] Herdman M, Gudex C, Lloyd A et al. Development and preliminary testing of the new five-level version of EQ-5D (EQ-5D-5L). Quality of Life Research [Internet] Springer. 2011 [cited 2024 Nov 14];20:1727–1736. Available from: https://link.springer.com/article/10.1007/s11136-011-9903-x 10.1007/s11136-011-9903-x PMC3220807 21479777

[18] Jones PW, Harding G, Berry P et al. Development and first validation of the COPD Assessment Test. Eur Respir J [Internet] Eur Respir J. 2009 [cited 2024 Nov 14];34:648–654. Available from: https://pubmed.ncbi.nlm.nih.gov/19720809/10.1183/09031936.00102509 19720809

[19] Kon SSC, Canavan JL, Jones SE et al. Minimum clinically important difference for the COPD assessment test: a prospective analysis. Lancet Respir Med [Internet] Lancet Publishing Group. 2014 [cited 2024 Nov 13];2:195–203. Available from: http://www.thelancet.com/article/S2213260014700013/fulltext 10.1016/S2213-2600(14)70001-3 24621681

[20] Sun S, Chuang LH, Sahlén KG et al. Estimating a social value set for EQ-5D-5L in Sweden. Health qual life outcomes [Internet] BioMed Central Ltd. 2022 [cited 2024 Nov 13];20:1–12. Available from: https://hqlo.biomedcentral.com/articles/10.1186/s12955-022-02083-w 10.1186/s12955-022-02083-w PMC9780618 36564844

[21] Nolan CM, Longworth L, Lord J et al. The EQ-5D-5L health status questionnaire in COPD: validity, responsiveness and minimum important difference. Thorax [Internet] BMJ Publishing Group Ltd. 2016 [cited 2024 Nov 13];71:493–500. Available from: https://thorax.bmj.com/content/71/6/493 10.1136/thoraxjnl-2015-207782 PMC4893131 27030578

[22] R Core Team. R: A language and environment for statistical computing [Internet]. Vienna, Austria. 2024. Available from: https://www.R-project.org/

[23] Kvale PA, Conway WA, Coates EO. Continuous or nocturnal oxygen therapy in hypoxemic chronic obstructive lung disease. a clinical trial. Ann Intern Med. 1980;93:391–8. 6776858 10.7326/0003-4819-93-3-391

[24] Hardinge M, Annandale J, Bourne S, BMJ Publishing Group Ltd. British thoracic society guidelines for home oxygen use in adults: accredited by NICE. Thorax [Internet]. 2015 [cited 2025 Jan 22];70:i1–i43. Available from: https://thorax.bmj.com/content/70/Suppl_1/i1 10.1136/thoraxjnl-2015-206865 25870317

[25] Drummond MB, Wise RA. Oxygen therapy in COPD: What do we know? https://doi.org/101164/rccm200705-682ED [Internet] American Thoracic Society. 2012 [cited 2025 Jan 22];176:321–322. Available from: https://www.atsjournals.org. 10.1164/rccm.200705-682ED 17675448

[26] Gloeckl R, Marinov B, Pitta F. Practical recommendations for exercise training in patients with COPD. European Respiratory Review[Internet] European Respiratory Society. 2013 [cited 2025 Jan 22];22:178–186. Available from: https://publications.ersnet.org/content/errev/22/128/178 10.1183/09059180.00000513 PMC9487382 23728873

[27] Venkatesan P, GOLD, COPD report.: 2024 update. Lancet Respir Med [Internet] Lancet Respir Med. 2024 [cited 2025 Jan 22];12:15–16. Available from: https://pubmed.ncbi.nlm.nih.gov/38061380/10.1016/S2213-2600(23)00461-7 38061380

[28] Paneroni M, Ambrosino N, Simonelli C et al. Physical activity in patients with chronic obstructive pulmonary disease on long-term oxygen therapy: a cross-sectional study. Int J Chron Obstruct Pulmon Dis [Internet] Int J Chron Obstruct Pulmon Dis. 2019 [cited 2025 Jan 22];14:2815–2823. Available from: https://pubmed.ncbi.nlm.nih.gov/31824146/10.2147/COPD.S228465 PMC6901041 31824146

[29] Sanchez-Morillo D, Muñoz-Zara P, Lara-Doña A et al. Automated home oxygen delivery for patients with COPD and respiratory failure: a new approach. Sensors. [Internet] Multidisciplinary digital publishing institute. 2020 [cited 2025 Jan 22];20:1178. Available from: https://www.mdpi.com/1424-8220/20/4/1178/htm 10.3390/s20041178 PMC7070269 32093418

[30] Mól CG, da Silva Vieira AG, Garcia BMSP et al. Closed-loop oxygen control for critically ill patients––a systematic review and meta-analysis. PLoS One [Internet] Public library of science. 2024 [cited 2025 Jan 22];19:e0304745. Available from: https://pmc.ncbi.nlm.nih.gov/articles/PMC11168613/10.1371/journal.pone.0304745 PMC11168613 38865428

[31] Katsenos S, Constantopoulos SH. Long-term oxygen therapy in copd: factors affecting and ways of improving patient compliance. Pulm Med [Internet] Hindawi publishing corporation pulmonary medicine. 2011 [cited 2025 Jan 22];2011:325362. Available from: https://pmc.ncbi.nlm.nih.gov/articles/PMC3175397/10.1155/2011/325362 PMC3175397 21941649

[32] Jacobs SS, Lederer DJ, Garvey CM et al. Optimizing home oxygen therapy. An official american thoracic society workshop report. Ann Am Thorac Soc [Internet] Ann Am Thorac Soc. 2018 [cited 2025 Jan 22];15:1369–1381. Available from: https://pubmed.ncbi.nlm.nih.gov/30499721/10.1513/AnnalsATS.201809-627WS 30499721

[33] Jones PW, Rennard S, Tabberer M et al. Interpreting patient-reported outcomes from clinical trials in COPD: a discussion. Int J Chron Obstruct Pulmon Dis [Internet] Dove Medical Press Ltd. 2016 [cited 2025 Jan 22];11:3069. Available from: https://pmc.ncbi.nlm.nih.gov/articles/PMC5153282/10.2147/COPD.S117378 PMC5153282 27994447

[34] Jeffrey AA, Ray S, Douglas NJ. Accuracy of inpatient oxygen administration. Thorax [Internet] Thorax. 1989 [cited 2025 Jan 31];44:1036–1037. Available from: https://pubmed.ncbi.nlm.nih.gov/2617443/10.1136/thx.44.12.1036 PMC1020881 2617443

[35] Tikellis G, Hoffman M, Mellerick C et al. Barriers to and facilitators of the use of oxygen therapy in people living with an interstitial lung disease: a systematic review of qualitative evidence. European Respiratory Review [Internet] European Respiratory Society; 2023 [cited 2025 Jan 22];32:230066. Available from: https://pmc.ncbi.nlm.nih.gov/articles/PMC10445108/10.1183/16000617.0066-2023 PMC10445108 37611946

