# Supplementary material for: FlexO2: A patient-controlled oxygen flow selector improving autonomy and daily function in long-term oxygen therapy (LTOT)
Source: Respir Res. 2025 May 26;26:200. doi: 10.1186/s12931-025-03274-x (PMC12107881; doi:10.1186/s12931-025-03274-x)
Supplement: Supplementary file 1 — Supplementary Material 1 [file 12931_2025_3274_MOESM1_ESM.docx]

**Supplementary file**

**Table S1**

Table S1. Oxygen Dose Adjustments During Daily Activities at Baseline and 3-Month Follow-up with FlexO2 Device Use

| **Situations** | **Baseline,** N = 26 | | **3 Months,** N = 26 | | **p-value^a^** |
| --- | --- | --- | --- | --- | --- |
|  | **N** | **Percentage** | **N** | **Percentage** |  |
| **When getting out of bed** | 8 | 31% | 21 | 81% | **<0.001** |
| **When getting up from sitting to standing** | 4 | 15% | 16 | 62% | **0.002** |
| **With moving around at home** | 10 | 40% | 22 | 88% | **0.003** |
| Missing N (%) | 1 | 3.8% | 1 | 3.8% |  |
| **With daily hygiene** | 13 | 50% | 20 | 80% | 0.07 |
| Missing N (%) | 0 | 0% | 1 | 3.8% |  |
| **With dressing and undressing** | 8 | 31% | 23 | 88% | **<0.001** |
| **With household chores** | 12 | 50% | 20 | 95% | **0.013** |
| Missing N (%) | 2 | 7.7% | 5 | 19% |  |
| **With meals** | 2 | 8.3% | 11 | 46% | **0.027** |
| Missing N (%) | 2 | 7.7% | 2 | 7.7% |  |
| **With exercise** | 14 | 67% | 16 | 94% | 0.4 |
| Missing N (%) | 5 | 19% | 9 | 35% |  |
| **With other physical activity** | 13 | 57% | 19 | 86% | **0.023** |
| Missing N (%) | 3 | 12% | 4 | 15% |  |
| **With shortness of breath** | 14 | 54% | 12 | 92% | **0.008** |
| Missing N (%) | 0 | 0% | 1 | 3.8% |  |
| **When going to the toilet** | 7 | 27% | 18 | 72% | **0.003** |
| Missing N (%) | 0 | 0% | 1 | 3.8% |  |
| **When walking up stairs** | 11 | 58% | 10 | 91% | 0.2 |
| Missing N (%) | 7 | 27% | 15 | 58% |  |
| **Total^b^** |  | |  | |  |
| Median (Q1, Q3) | 5.0 (1.0, 7.0) | | 9.0 (7.0, 10.0) | | **<0.001** |
| ^a^ P-value was calculated using the Wilcoxon signed-rank test for paired continuous data, and McNemar's test was used for paired categorical data  ^b^ Total number of situations per patient in which oxygen dose adjustment was needed. | | | | | |

**Table S2**

Table S2. Adherence at Baseline and 3-Month Follow-up with FlexO2 Device Use

|  | **Baseline,**  N = 26 | **3 Months,**  N = 26 | **p-value^a^** |
| --- | --- | --- | --- |
| Has it been easy to follow the prescribed dose? | 14 (54%) | 22 (85%) | **0.043** |
| ^a^ McNemar's test was used for paired categorical data | | | |

**-**
